# Happier People Show Greater Neural Connectivity during Negative Self-Referential Processing

**DOI:** 10.1371/journal.pone.0149554

**Published:** 2016-02-22

**Authors:** Eun Joo Kim, Sunghyon Kyeong, Sang Woo Cho, Ji-Won Chun, Hae-Jeong Park, Jihye Kim, Joohan Kim, Raymond J. Dolan, Jae-Jin Kim

**Affiliations:** 1 Graduate School of Education, Yonsei University, Seoul, Republic of Korea; 2 Institute of Behavioral Science in Medicine, Yonsei University College of Medicine, Seoul, Republic of Korea; 3 Department of Nuclear Medicine, Yonsei University College of Medicine, Seoul, Republic of Korea; 4 Department of Psychology, Yonsei University, Seoul, Republic of Korea; 5 Department of Communication, Yonsei University, Seoul, Republic of Korea; 6 Wellcome Trust Centre for Neuroimaging at UCL, London, United Kingdom; 7 Department of Psychiatry, Yonsei University Gangnam Severance Hospital, Seoul, Republic of Korea; University of Groningen, NETHERLANDS

## Abstract

Life satisfaction is an essential component of subjective well-being and provides a fundamental resource for optimal everyday functioning. The goal of the present study was to examine how life satisfaction influences self-referential processing of emotionally valenced stimuli. Nineteen individuals with high life satisfaction (HLS) and 21 individuals with low life satisfaction (LLS) were scanned using functional MRI while performing a face-word relevance rating task, which consisted of 3 types of face stimuli (self, public other, and unfamiliar other) and 3 types of word stimuli (positive, negative, and neutral). We found a significant group x word valence interaction effect, most strikingly in the dorsal medial prefrontal cortex. In the positive word condition dorsal medial prefrontal cortex activity was significantly higher in the LLS group, whereas in the negative word condition it was significantly higher in the HLS group. The two groups showed distinct functional connectivity of the dorsal medial prefrontal cortex with emotional processing-related regions. The findings suggest that, in response to emotional stimuli, individuals with HLS may successfully recruit emotion regulation-related regions in contrast to individuals with LLS. The difference in functional connectivity during self-referential processing may lead to an influence of life satisfaction on responses to emotion-eliciting stimuli.

## Introduction

Human experience is strongly influenced by how much external stimuli are perceived as self-related. When stimuli are viewed as self-referential, associated neural activity can index a conscious reflection on oneself, which is referred to as self-referential processing (SRP) [1]. Previous studies have shown a specific pattern of neural activity during various expressions of SRP. For example, recognition of one’s own face elicits activity in brain regions that is distinct from activity evoked by other-faces, and these regions include the medial prefrontal cortex (MPFC), anterior cingulate cortex (ACC), posterior cingulate cortex (PCC), precuneus, and anterior insular cortex [2,3]. Based on these results, the existence of a self-specific network has been proposed [4]. A previous study has shown that a functional response within this network is more pronounced during processing of negative stimuli than positive ones [5].

There is an emerging consensus that the MPFC is strongly linked to self-related information processing [6,7]. In terms of self-relatedness a functional dissociation between the dorsal and ventral portions of the MPFC is proposed, though this distinction remains controversial [8–11]. Indeed there is evidence that the role of the MPFC in SRP may also relate to a specific contribution in human social regulations. For example, MPFC activity has been shown to reflect processing based upon attending either to one's own or others’ emotions and mental states [12] as well as a simulation of possible behaviors of others rather than actuated behavior [13]. This region is also associated with a focusing of attention on judgments about external stimuli as well as supporting reflective processes for selecting higher level of social and affective meanings [14]. On this basis the MPFC has been considered to be a module that integrates social information across time [15].

SRP may be related to life satisfaction in that it is an essential component of subjective well-being, defining how people evaluate their lives–both in the moment and over extended periods of time [16]. Life satisfaction is also fundamental for optimal functioning [17], including positive life experience and accomplishments [17,18]. Individuals with higher life satisfaction reported greater appreciation for hypothetical rewarding events [19], greater flexibility and adjustment to negative feedback [20,21], and more positive self-cognitions [22,23]. In addition, individuals with higher life satisfaction are relatively insensitive to negative stimuli [24,25] and react more positively to contexts [25,26]. Although a neural correlate of life satisfaction is unclear and there is little research that addresses this question, a recent report has emphasized the role of the amygdala and other emotion processing regions in an influence of life satisfaction [27].

One of the most widely investigated individual differences in the domain of cognition research is self-esteem rather than life satisfaction. For instance, individuals with low self-esteem who experience greater levels of social pain have greater dorsal ACC activity [28] and rostral ACC activation which is different according to the level of attentional control [29]. There is a report that self-esteem is associated with hippocampal volume and the cortisol response to a psychosocial stress [30]. In terms of SRP, women with lower self-esteem show greater activation in the ventral MPFC and ACC during negative SRP, while women who experience greater positive affect during positive SRP show greater dorsal MPFC activation [31]. Self-esteem modulates MPFC or ACC activity in response to evaluative social feedback [32,33], as well as PCC activity during processing of positive self-face evaluation as self-referential stimuli [34]. The present functional magnetic resonance imaging (fMRI) study extends on this previous SRP research by examining the role of life satisfaction as opposed to self-esteem. Given that SRP might concern not only verbal stimuli but also autobiographical, emotional, motor, and facial stimuli [1], we developed a relevance rating task using a combination of faces and words for addressing positive and negative SRP.

In fact, self-esteem is the strongest predictor of life satisfaction [24], and there is a critical difference between high and low life satisfaction in determining the level of self-esteem [16]. Nonetheless, self-esteem and life satisfaction though highly intercorrelated are distinctly constructed [18,24,35]. As is the case for self-esteem, life satisfaction might be expected to influence how an individual processes positive or negative external stimuli [36], though relevant research is sparse (see S1 Table). Since life satisfaction plays such a vital role in subjective well-being and quality of life, it is important to understand how life satisfaction influences a range of psychological process particularly at a neural level.

In the present fMRI study, we investigated neural activation and connectivity patterns during SRP related to individual differences in life satisfaction. SRP was addressed using the face-word relevance rating task, which we developed to examine how life satisfaction exerts an influence on SRP in response to presentation of emotionally valenced stimuli of self versus other. Consistent with recent studies, we expected that higher life satisfaction would be related to distinct patterns of neural connectivity as subjects process valenced stimuli, particularly within regions related to SRP. In particular, we predicted a key role for the MPFC and its connected regions in life satisfaction-related emotional processing.

## Materials and Methods

### Participants

Participants were recruited through advertisement at a local hospital and via the internet. Forty subjects (age: 38.25 ± 5.86 years, range 29–48; 20 females and 20 males) agreed to participate in the experiment that involved both an fMRI scan and assessment of SRP. All participants were right-handed, as assessed by the Annett Handedness Inventory [37]. Exclusion criteria included the presence of a neurological, psychiatric or significant medical illness, and a history of current or past substance abuse or dependence. The study was approved by the institutional review board of Yonsei University Severance Hospital, and written informed consent was obtained from all participants before the study began.

No participant was excluded from analysis. Life satisfaction was measured before the MRI procedure using the Satisfaction with Life Scale (SWLS) [38], the most widely-used self-report questionnaire designed to assess cognitive-evaluative aspects of subjective well-being. This scale has been proved to have good reliability and validity [39]. Participants rated how much they agree or disagree with five life satisfaction statements on 7-point Likert-type scales. Scores were summed to generate a total score ranging from 5 to 35. Given that the SWLS scores did not normally distribute (Kolmogorov-Smirnov normality test, p = 0.036) as shown in S1 Fig, we divided participants into two groups rather than treating them as a single group [40]. Based on median split (SWLS = 21), participants were divided into a high life satisfaction (HLS) group (n = 21) and a low life satisfaction (LLS) group (n = 19).

### Experimental Paradigm

**Fig 1:**
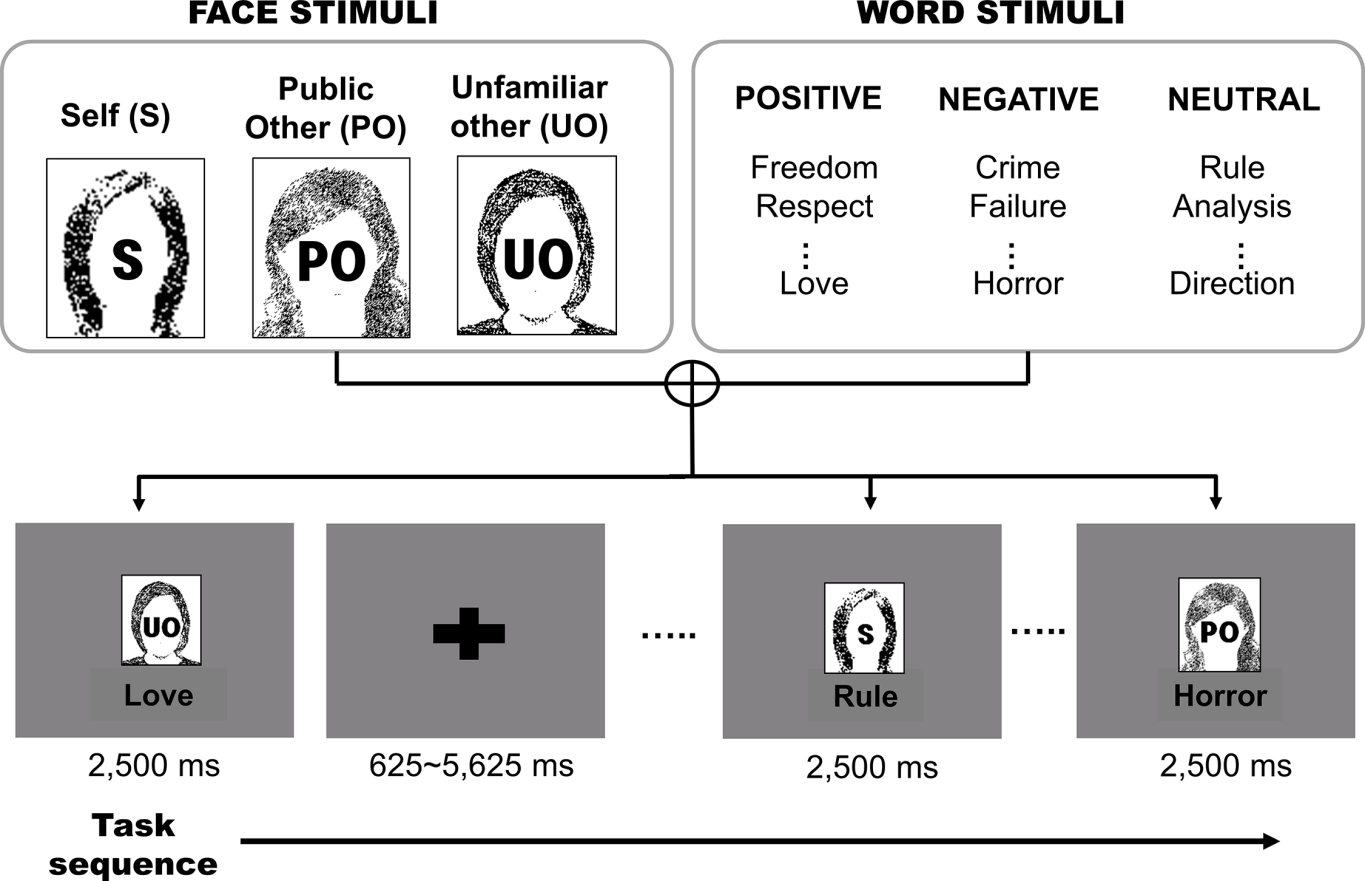
Trial sequence of the face word relevance rating task. The real face photos and Korean words were presented during fMRI experiment; in this figure, the visual stimuli are replaced with cartoon images and English words to help understand the task procedure.

During fMRI scanning participants performed the face-word relevance rating task, in which they were asked to view a stimulus consisting of a face in the upper position and a word in the lower position of a visual presentation (see Fig 1). A face stimulus was one of 3 types (self, public other, and unfamiliar other) and a word stimulus had one of 3 valences (positive, negative, and neutral), giving 9 different possible conditions. Each of these conditions was repeated 30 times, giving a total of 270 trials. A face stimulus presented one of 5 different persons across 3 types; a “self-face” picture, a “public other” comprising two pictures of famous Korean athletes (male 1, female 1), and an “unfamiliar other” comprising two pictures (male 1, female 1) selected from Korean Facial Expressions of Emotion [41]. All facial stimuli expressed only a neutral emotion. For word stimuli we used 270 nouns (90 positive, 90 negative, and 90 neutral). Each trial was presented for 2,500 ms duration, while null trials varied from 625 ms to 5,625 ms. The order of presentation was randomized and counterbalanced across participants. Participants’ task was to assess the relevance of the given word to the corresponding face and then press an appropriate button as quickly as possible (1 = irrelevant, 2 = neither relevant nor irrelevant, 3 = relevant).

The relevance rating and reaction time were automatically counted and used for statistical analyses. In order to verify the validity of the word stimuli, after fMRI scanning, participants were instructed to rate a questionnaire for valence of the presented words (very positive, 4; neutral, 0; and very negative, -4).

### MRI Data Acquisition and Preprocessing

A 3T MR scanner (Philips Medical system, Best, The Netherlands) was used in this study. Functional images were acquired using a T2*-weighted gradient echo echo-planar imaging sequence (34 slices of 4.5mm thickness and no gaps, repetition time [TR] = 2,500 ms, echo time [TE] = 35 ms, flip angle [FA] = 90°, image matrix = 128 x 128, field of view [FOV] = 220 mm) with an in-plane resolution of 1.719 mm x 1.719 mm. Structural images with a resolution of 0.859 mm x 0.859 mm x 1.2 mm were acquired using a 3D T1-weighted gradient echo sequence (195 slices, TR = 9.67 ms, TE = 4.60 ms, image matrix = 256 x 256).

Preprocessing and data analysis were conducted within SPM8 (Statistical Parametric Mapping, Wellcome Department of Cognitive Neurology, London, UK). Corrections for differences in slice acquisition time were performed, and then head motion was corrected by realignment. The corrected images were co-registered with the segmented T1-weighted image for each subject. The T1-weighted image was normalized to the standard T1 template, and then the resulting transformation matrices were applied to the co-registered functional images. These data were smoothed with a Gaussian kernel of 8-mm full-width at half-maximum.

### Statistical Analysis

Demographic information, clinical data and post-task valence ratings were compared between the groups using Student’s *t-*tests or Chi-square test. The behavioral responses were analyzed for the main effects of group, face and word valence and the interactions between them using repeated-measures ANOVA. *Post-hoc* analysis was performed to compare the behavioral responses between the HLS and LLS groups for each face and word valence condition using Student’s *t-*tests.

Preprocessed functional data were analyzed using a general linear model. Experimental trials were modeled separately using a canonical hemodynamic response function for individual data. Multiple linear regression, as implemented in SPM8 using a least-squares approach, was used to obtain parameter estimates. These parameter estimates were then further analyzed by testing specific contrasts using the participant as a random factor. Contrast images for self–minus-unfamiliar-other and public-other-minus-unfamiliar-other in each word condition were created for each participant on the first-level analysis. Individual realignment parameters were entered as regressors to control for movement-related variance. The contrast images were entered into the one-sample t-test in each group and the full factorial model across the participants. The one-sample t-test in each group was performed to find brain activations in each word valence condition, which were defined as a significant increase for self compared with public other. Statistical inferences were conducted at a threshold of AlphaSim corrected p < 0.05, which corresponded to a voxel-level threshold *p* < 0.001 and cluster size *k* > 36 voxels. The cluster size was determined through a Monte Carlo simulation using AFNI’s AlphaSim program (http://afni.nimh.nih.gov/afni/doc/manual/AlphaSim) with 10,000 iterations. In addition, in order to provide additional information, we conducted non-parametric spearman’s correlation analysis between the SWLS score and brain activity across all participants. In the full factorial model, activation maps for the main effects and interactions were analyzed as a 2 (group; HLS and LLS) x 2 (word valence; positive and negative) design in the same threshold with the one-sample t-test in each group. As a *post-hoc* test, the mean beta values in the spheres with a center of peak coordinate showing a significant group x word valence interaction and a 4-mm diameter were compared in each word condition using Student’s t-test at a significance of *p* < 0.05.

### Analysis for Interregional Functional Connectivity

We performed psychophysiological interaction (PPI) analysis [42] to identify modulation of functional connectivity in an identified SRP pathway as a function of word stimuli type. Specifically, context-dependent functional connectivity was estimated between a source and targets during the viewing of face stimuli in each word valence condition. Based on the previous research on the role of the MPFC in SRP [8–11], the right dorsal MPFC (x = 2, y = 24, z = 44) among significant clusters in the interaction effect of group and emotion was considered to be the source region of 4-mm sphere. Based on previous studies demonstrating functional connections of the MPFC for various processes of emotion [11,28,43–45], we defined eleven target regions of 4-mm sphere: the right dorsal and ventral MPFC, right dorsal and ventral ACC, left PCC, bilateral precentral gyri, bilateral temporo-parietal junction (TPJ), left insula, and left amygdala. Their coordinates are listed in S2 Table. Dorsal MPFC activity was the physiological regressor, whereas the face stimulus condition (self versus public other) was the psychological regressor. A third regressor represented the interaction between these two regressors. The psychological variable used was a vector coding for the specific task (1 for self, -1 for public other) convolved with the hemodynamic response function, and the physiological factor was then multiplied with the psychological factor to constitute the interaction term. PPI analyses were conducted for each subject with a design matrix including the three regressors. Individual contrast images were then entered into group analysis for each of the 4 subgroups such as HLS with positive words, HLS with negative words, LLS with positive words, and LLS with negative words.

## Results

### Demographic Information

The mean scores of the SWLS were 16.0 ± 3.2 (ranges, 10–20) in the LLS group and 23.6 ± 1.6 (ranges, 21–27) in the HLS group, and were significantly different between the two groups (t_38_ = 9.6, p < 0.001). There were no significant group differences either for gender (10 males in both groups), age (37.1 ± 5.9 years and 39.6 ± 5.7 years, respectively) and education (16.6 ± 2.1 years and 16.6 ± 3.8 years, respectively).

### Behavioral Responses

Table 1 reports the relevance rating and response time during the task in the HLS and LLS groups. The relevance rating showed a significant main effect of face (*F*_1,38_ = 51.3, *p* < 0.001) and word valence (*F*_1,38_ = 69.7, *p* < 0.001) and a significant face × word valence interaction (*F*_1,38_ = 51.3, *p* < 0.001). However, there were no significant main effect of group and interactions of group × face, group × word valence and group × face × word valence in the relevance rating. In *post-hoc* analysis, the only significant group difference was observed in the self and neutral word condition where the relevance rating was significantly higher in the HLS group than in the LLS group (p<0.05). The response time also showed a significant main effect of face (*F*_1,38_ = 42.9, *p* < 0.001) and word valence (*F*_1,38_ = 25.6, *p* < 0.001), and a significant face × word valence interaction (*F*_1,38_ = 25.6, *p* < 0.001), but no significant main effect of group and interactions of group × face, group × word valence and group × face × word valence. In *post-hoc* analysis the response time showed no group difference in any face and word valence condition. In the post-scanning word valence rating, the HLS and LLS groups showed no difference in their rating of positive (2.8 ± 0.7, 2.6 ± 0.8, respectively), neutral (1.1 ± 0.7, 0.8 ± 0.7, respectively), and negative (-2.4 ± 0.9, -2.4 ± 0.8, respectively) words.

**Table 1:** The relevance rating and response time (mean and standard deviation) as a task performance in the high life satisfaction (HLS) and low life satisfaction (LLS) groups.

	Relevance rating			Response time (ms)		
Event	HLS	LLS	P-value	HLS	LLS	P-value
Positive words						
Self	2.74 (0.40)	2.52 (0.47)	.076	963.24 (209.70)	1040.37 (195.54)	.114
Public other	2.75 (0.35)	2.63 (0.34)	.177	1001.28 (162.22)	1079.61 (209.8)	.110
Unfamiliar other	2.04 (0.61)	1.96 (0.38)	.326	1142.17 (180.64)	1182.13 (166.83)	.221
Neutral words						
Self	2.42 (0.37)	2.16 (0.36)	.032	1132.71 (195.59)	1175.46 (199.43)	.480
Public other	2.40 (0.42)	2.33 (0.33)	.422	1158.67 (159.76)	1217.91 (203.17)	.261
Unfamiliar other	1.87 (0.42)	1.79 (0.23)	.258	1156.94 (156.51)	1199.06 (206.17)	.490
Negative words						
Self	1.25 (0.24)	1.25 (0.19)	.682	1032.06 (142.82)	1060.76 (168.27)	.305
Public other	1.20 (0.22)	1.33 (0.32)	.099	984.27 (250.7)	1116.43 (204.48)	.080
Unfamiliar other	1.34 (0.39)	1.33 (0.27)	.823	1082.37 (181.81)	1097.81 (179.71)	.498

### Brain Activation during SRP as a Function of Face-word Condition and Life Satisfaction Grouping

As shown in Table 2, a network of brain regions were activated while seeing self faces compared with seeing public other faces under each word valence condition. For the positive word condition, significant effects were seen in the left middle temporal gyrus, right hippocampus, left thalamus, and right caudate in the HLS group, whereas in the LLS group activations were seen in prefrontal regions, including the left dorsal MPFC and parietal regions. For the negative word condition, in the HLS group enhanced activity was seen in regions including the right dorsal MPFC, left DLPFC, right superior frontal gyrus, bilateral TPJ, left precuneus, and right cerebellum, but no effects were found for the same contrast in the LLS group. Likewise, for the neutral word condition, the LLS group showed no activation, but the HLS group showed significant activations in the left dorsal MPFC, left superior frontal gyrus, right precentral gyrus, right posterior cingulate cortex, left caudate, and left cerebellum. Meanwhile, results from the correlation analysis between the SWLS score and brain activity across all participants were summarized in the supplementary material (S3 Table).

**Table 2:** Brain activation in each word valence condition while seeing self compared with public other in each life satisfaction group.

	High life satisfaction group					Low life satisfaction group				
	Coordinate, mm					Coordinate, mm				
	x	y	z	*k*	Zmax	x	y	z	*k*	Zmax
<Positive words>										
L. Dorsal MPFC						-2	50	30	1093	4.68
						-12	28	58	350	4.33
L. Ventral MPFC						-8	64	4	120	4.71
R. Superior frontal gyrus						16	40	54	71	3.94
L. Inferior frontal gyrus						-54	20	10	49	4.11
R. PCC						2	-24	26	59	3.78
L. TPJ						-50	-46	28	96	3.71
L. Middle temporal gyrus	-36	-60	14	486	4.88					
R. Hippocampus	38	-46	6	240	4.00					
L. Thalamus	-10	-24	14	116	3.72					
R. Caudate	22	24	8	53	3.69					
<Negative words>										
R. Dorsal MPFC	6	14	66	75	3.78					
L. DLPFC	-26	36	40	62	3.57					
R. Superior frontal gyrus	20	60	28	158	4.08					
L. TPJ	-50	-54	36	114	4.25	No significant results				
R. TPJ	52	-52	44	85	3.65					
L. Precuneus	-12	-70	32	42	3.57					
R. Cerebellum	4	-78	-22	198	4.06					
<Neutral words>										
L. Dorsal MPFC	0	36	26	122	3.82					
L. Superior frontal gyrus	-24	44	40	99	3.81					
R. Precentral gyrus	40	10	28	183	4.38					
R. PCC	4	-22	40	126	3.96	No significant results				
L. Caudate	-4	4	4	62	4.30					
L. Cerebellum	-18	-76	-14	123	3.92					
	-8	-60	-18	69	3.74					

Statistical threshold at AlphaSim-corrected *P*<0.05

L., left; R., right; MPFC, medial prefrontal cortex; PCC, posterior cingulate cortex; TPJ, temporo-parietal junction; DLPFC, dorsolateral prefrontal cortex.

### SRP-related Responses According to Life Satisfaction and Word Valence

Table 3 presents an examination of group (HLS versus LLS) and word valence (positive versus negative) factors while seeing self-faces compared with public other faces. A main effect of group was seen in the right angular gyrus. A main effect of word valence was observed in the right angular gyrus, right posterior cingulate cortex, left thalamus, right putamen, and right caudate.

**Table 3:** The main and interaction effects of group and word valence while seeing self compared with public other.

Regions	*k*	*F*	Zmax	Coordinate, mm		
				x	y	z
**Main effect of group** (high satisfaction versus low satisfaction)						
R. Angular gyrus	46	18.62	3.90	36	-48	34
L. Cerebellum	71	16.91	3.72	-8	-66	-50
**Main effect of word valence** (positive versus negative)						
R. Angular gyrus	37	18.50	3.89	38	-44	24
R. Posterior cingulate cortex	52	14.53	3.45	0	-52	22
L. Thalamus	132	19.39	3.98	-10	-26	16
R. Putamen	77	16.93	3.73	28	-4	10
R. Caudate	210	27.92	4.72	20	-30	16
**Interaction effect by group x word valance**						
R. DLPFC	80	20.91	4.13	30	24	48
L. Dorsal MPFC	98	16.57	3.69	-8	26	56
R. Dorsal MPFC		15.64	3.58	2	24	44

Statistical threshold at AlphaSim-corrected *P*<0.05

L., left; R., right; MPFC, medial prefrontal cortex; DLPFC, dorsolateral prefrontal cortex.

**Fig 2:**
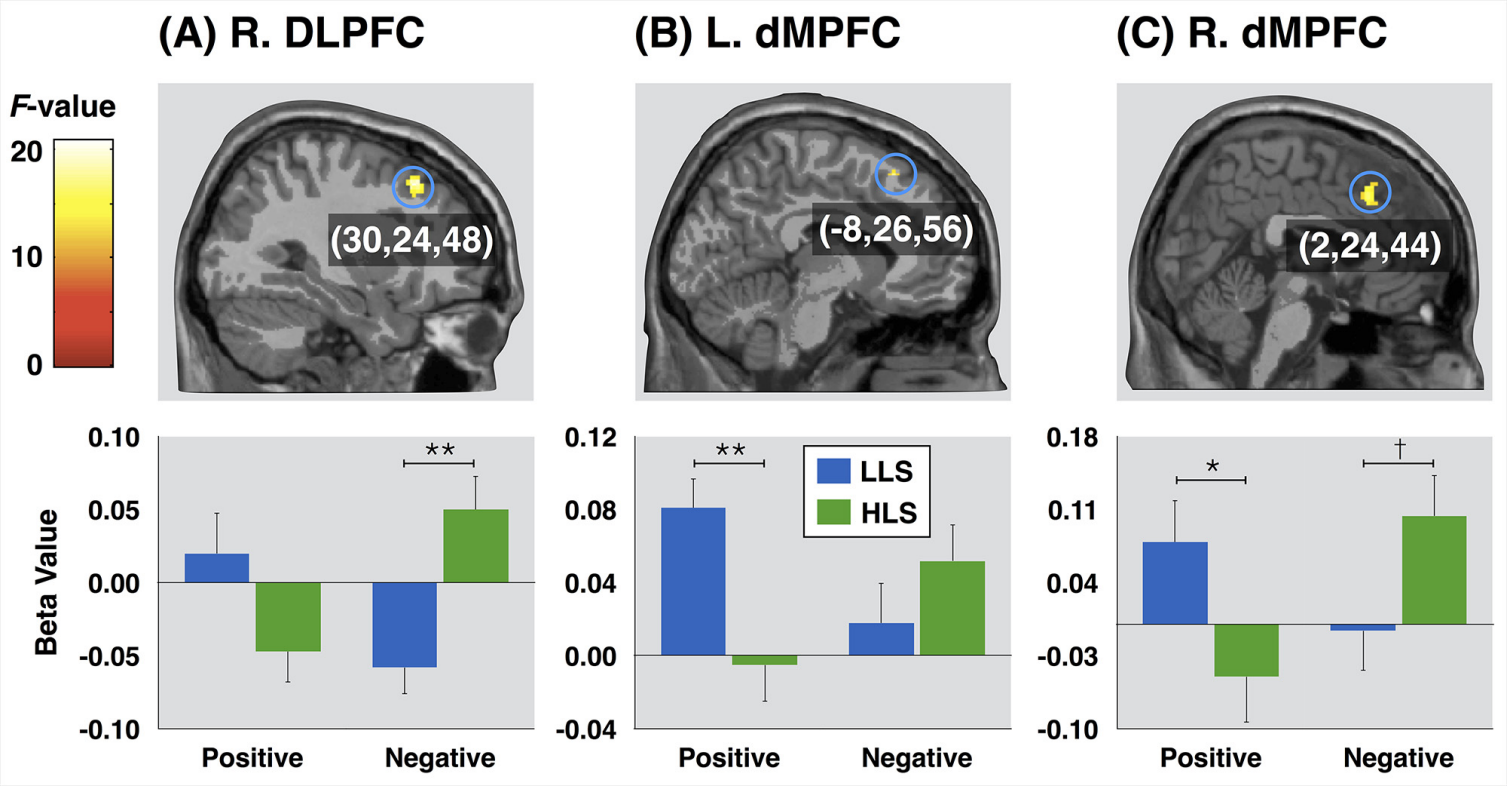
Brain regions showing the interaction effect between group and word valence and *post-hoc* analyses for group differences (**p<0.01; *p<0.05; and †p = 0.05). Error bars represent standard errors. R., right; L., left; DLPFC, dorsolateral prefrontal cortex; dMPFC, dorsal medial prefrontal cortex; LLS, low life satisfaction group; and HLS, high life satisfaction group.

A group x word valence significant interaction effect was seen in the right dorsolateral prefrontal cortex (DLPFC) and bilateral dorsal MPFC. The results from a *post-hoc* analysis on these regions to characterize the interaction are shown in Fig 2. Beta values in the right DLPFC in the positive word condition did not significantly differ between the two groups, whereas those in the negative word condition were significantly higher in the HLS group compared to the LLS group (t_38_ = 3.61, p < 0.01). Compared to the HLS group, the LLS group showed beta values in the left dorsal MPFC that were higher in the positive word condition (t_38_ = 3.31, p < 0.01), whereas in the negative word condition beta values did not differ between the two group. Similarly, in the positive word condition beta values in the right dorsal MPFC were significantly higher in the LLS group than in the HLS group (t_38_ = 2.15, p < 0.05), whereas in the negative word condition they were higher on a marginal significance in the HLS group than in the LLS group (t_38_ = 2.01, p = 0.05). In the neutral word condition, beta values in all regions were not significantly different between the two groups.

### Interregional Functional Connectivity

**Fig 3:**
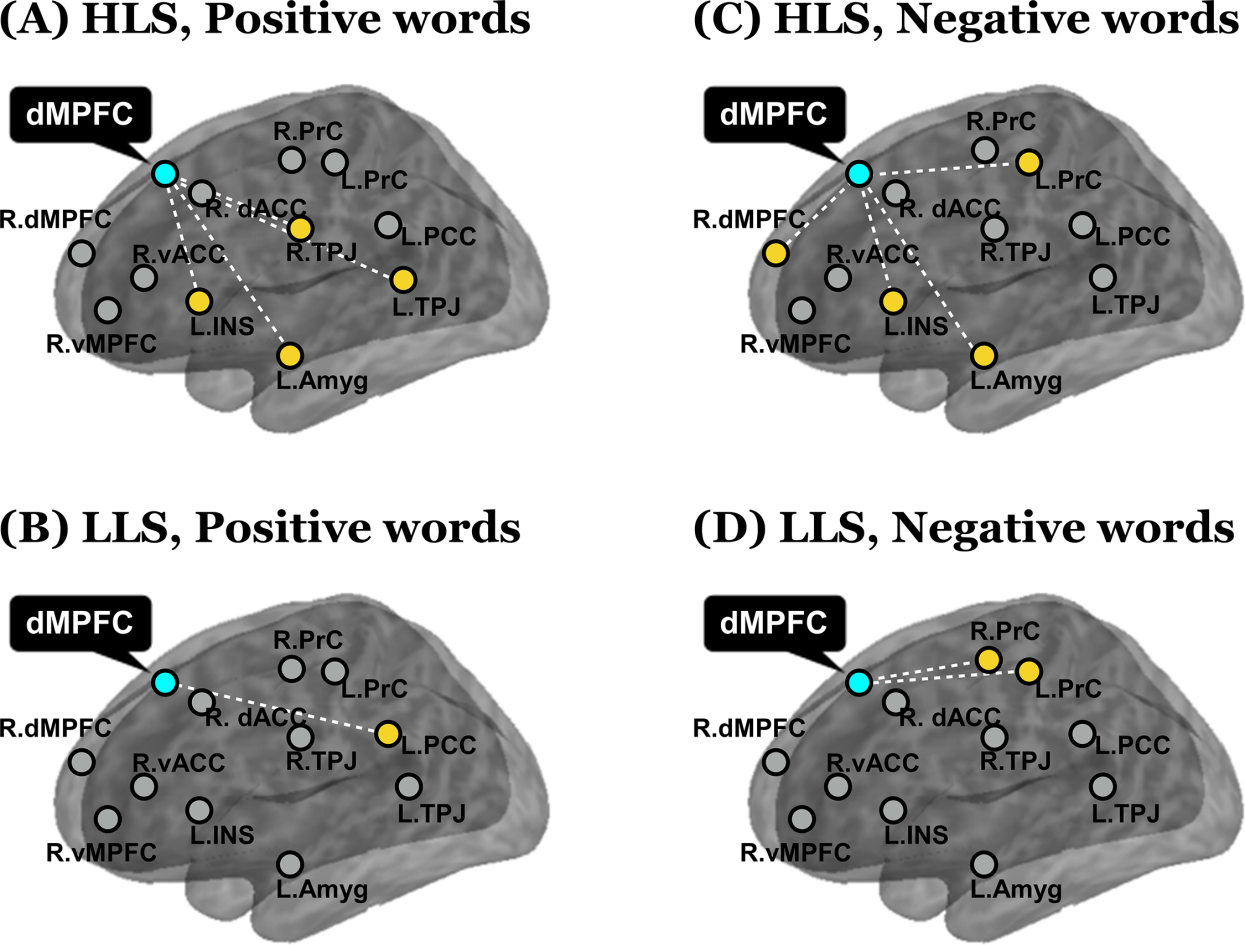
Context-dependent functional connectivity in the self when compared with the public other between a source (x = 2, y = 24, z = 44) in the dorsal medial prefrontal cortex (dMPFC) and *a priori* regions of interest in each life satisfaction group. The white dotted lines showed a significantly increased functional connectivity between the dMPFC and *a priori* targets. R. dMPFC, right dorsal medial prefrontal cortex; R. vMPFC, right ventral medial prefrontal cortex; R. dACC, right dorsal anterior cingulate cortex; R. vACC, right ventral anterior cingulate cortex; R. TPJ, right temporo-parietal junction; L. TPJ, left temporo-parietal junction; L. PCC, left posterior cingulate cortex; R. PrC, right precentral gyrus; L. PrC, left precentral gyrus; L. INS, left insula; and L. Amyg, left amygdala.

Focusing on the effects of positive and negative word conditions compared to the neutral word condition, we performed a PPI analyses to assess context-dependent functional connectivity between the seed region and *a priori* other regions. The right dorsal MPFC showing the significant group x word valence interaction was used as the seed region because it was considered to be a core region of SRP and revealed significant group differences in the opposite direction between the word valence conditions. Significantly increased functional connectivity is illustrated in Fig 3, and descriptively there was a greater number of increased functional connectivity in the HLS group compared to the LLS group.

In the positive word condition, the HLS group showed significantly increased functional connectivity of the dorsal MPFC with various regions including the left amygdala, left insula, and bilateral TPJ, whereas the LLS group revealed it only with the left PCC. In the negative word condition, the HLS group also showed significantly increased functional connectivity of the dorsal MPFC with various regions including the left amygdala, left insula, left precentral gyrus, and right dorsal MPFC, whereas the LLS group revealed it only with the bilateral precentral gyri. The HLS and LLS groups showed no overlapping significant functional connectivity in both positive and negative word conditions.

## Discussion

In the current study, the most prominent finding is a differential HLS and LLS group activation and connectivity profile in response to positive or negative stimuli. These imaging results are contrast with an absence of any difference in task performances between the two groups, except for relevance rating in the self and neutral word condition. The findings suggest that trait level life satisfaction modulates responses to emotional stimuli leading to distinct neural profiles during SRP.

As demonstrated in Fig 2, which depicts *post-hoc* results of the group-by-word valence interaction, the LLS group showed greater bilateral dorsal MPFC responses in the positive word condition than the HLS group. Conversely, in the negative word condition, the HLS group yielded a greater response in the right dorsal MPFC than the LLS group. Similar results are also demonstrated in Table 2, which shows word valence-related activations in each group. A previous study demonstrated that both up-regulation and down-regulation of positive emotion was associated with altered activity within the dorsal MPFC [46]. Although there was only marginal significance, lower relevance rating for self-face and positive words in the LLS group than in the HLS group invokes a possibility that dorsal MPFC activity reflects conflict between negative self evaluation and the positive emotion evoked by the latter stimuli. Meanwhile, given that the dorsal MPFC reflect cognitive demand for reappraisal in order to decrease negative emotion [47], our findings are consistent with the idea that individuals with higher life satisfaction invoke more effective regulatory processes in response to negative emotion than individuals with lower life satisfaction. This suggestion is consistent with a previous finding that individuals with higher life satisfaction think about negative stimuli in more positive and productive ways [25].

While seeing self compared to public other faces, the LLS group showed greater activation within the superior frontal gyrus and TPJ in the positive word condition while seeing self faces compared to public other faces, whereas the HLS group activated these two regions in the negative word condition. The superior frontal gyrus and TPJ are considered to be involved in processes related to psychological distancing, for example the process required when viewing an image from the perspective of a detached and distant observer [48,49], which appears to be helpful in reducing the intensity of negative emotion [50]. Within this framework, increased activation in these two regions might reflect a greater flexibility and adjustment to negative feedback in individuals with higher life satisfaction [20,21]. On the contrary, individuals with lower life satisfaction might distance themselves psychologically from positive stimuli, resulting in less positive view about their life experiences.

The DLPFC was activated for negative word stimuli in the HLS group, but not in the LLS group. This was also observed in the *post-hoc* test for the group x word valence interaction effect. This region is known to be activated by negative performance feedback [51], and a dysfunctional response of this region to negative stimuli has been observed in depressed individuals [52], suggesting an important contribution in an appropriate processing of negative information. Based on this our finding of DLPFC activation in response to negative words solely in individuals with higher life satisfaction suggests this region may be linked to processing self-related information in a more adaptive manner.

In a PPI analysis, the left amygdala and left insula were highly connected with the dorsal MPFC in the both positive and negative word conditions only in the HLS group. The amygdala has been reported to be related to life satisfaction [27] and to be correlated with the dorsal MPFC while trying to increase positive emotion [53]. Previous behavioral studies have shown that individuals with higher life satisfaction respond more positively to contexts [26,25]. The amygdala has also been found to be related to emotion regulation [54–56], and its involvement here is in keeping with our observation that emotional words evoked proper regulation in the HLS group, but not in the LLS group. Likewise, the connectivity with the insula in the HLS group is consistent with active emotion regulation as this region is part of the emotion-appraisal and regulatory system [57]. Previous studies have reported that depressed individuals show less emotion regulation-related connectivity between the amygdala and insula [55]. We can speculate that the HLS group maintains a high level of life satisfaction through increasing emotion regulation in response to the emotional word stimuli. It is of interest in this context that previous studies on life satisfaction have reported that individuals with higher life satisfaction are less sensitive to negative stimuli [24,25]. Our findings suggest that the insensitivity of individuals with higher life satisfaction toward the negative stimuli is an active neural process.

For positive stimuli the functional connection was significant only between dorsal MPFC and PCC in the LLS group. In a previous study, the correlation between these two regions was related to a self-referential condition compared with a non-referential condition [58]. Based on a previous finding that the PCC is involved in ambivalent evaluation [59], we speculate that individuals with lower life satisfaction may perceive the positive stimuli toward the self as ambivalent. As for the negative stimuli, the only significant connection in the LLS group was between the dorsal MPFC and precentral gyrus and we assume that this weaker connectivity of dorsal MPFC with other SRP-related regions is a neural signature of a behavioral feature of individuals with lower life satisfaction, specifically their failure to effectively manage negative information about the self.

We interpret group difference in functional connectivity between the dorsal MPFC and other *a priori* regions in terms of emotion regulation. Our findings indicate that a life satisfaction trait has a major impact on a response to positive or negative stimuli. Especially in an educational context, our findings suggest that the negative feedback to individuals with lower life satisfaction should be approached with sensitivity and caution.

There are some limitations in our study. As the majority of participants were in mid-30s to mid-40s, the study did not account for age variation both in life satisfaction and SRP. Furthermore, some confounding factors originated from the composition of the face-word relevance task might not be controlled in the analysis. First, in order to control the component of familiarity, two pictures of famous athletes were used in the control task. This means the comparison between self and public other could be biased by a component of valence. Second, in the task design, the task presentation time for face-word relevance rating in each trial was 2.5 sec, which might be too short for a deep reflection upon the self-referential nature of the word stimuli. The frequency of exposure to facial stimuli was different between the self and others, and thus there might be a different novelty effect between the conditions. Third, personality was not considered in the analysis, though previous studies have suggested that personality is an important predictor of life satisfaction [60,61]. In addition, only cognitive satisfaction with life was considered in the present study. It has been reported that the influence of personality on life satisfaction is mediated by hedonic balance and the relation between hedonic balance and life satisfaction is moderated by culture [62]. Therefore, the affective component of subjective well-being would have needed to be evaluated and considered in the analysis.

In conclusion, our study demonstrates that SRP, in response to emotional word stimuli, can be modulated by life satisfaction. In particular, the HLS group successfully recruited brain regions related to emotion regulation in contrast to the LLS group, suggesting that effectively regulating emotion might be a basis for higher life satisfaction. Additionally, the dorsal MPFC showed distinct connectivity pattern to positive and negative stimuli in the HLS and LLS groups, suggesting that this region might account for the difference in life satisfaction. These results can be applied in educational or working context to improve individual's response.

## Supporting Information

S1 Fig Distribution of the satisfaction with life scale scores in the low life satisfaction (LLS) and high life satisfaction (HLS) groups. (TIF)

S1 Table Previous neuroscience research on self-esteem and life satisfaction. (XLSX)

S2 Table Regions of interests used in psychophysiological interaction analysis. (XLSX)

S3 Table Spearman correlations between the satisfaction with life scale score and brain activity in each word valence condition while seeing self compared with public other faces. (XLSX)
