# Supplementary figures and images for: Happier People Show Greater Neural Connectivity during Negative Self-Referential Processing
Source: PLoS One. 2016 Feb 22;11(2):e0149554. doi: 10.1371/journal.pone.0149554 (PMC4763307; doi:10.1371/journal.pone.0149554)

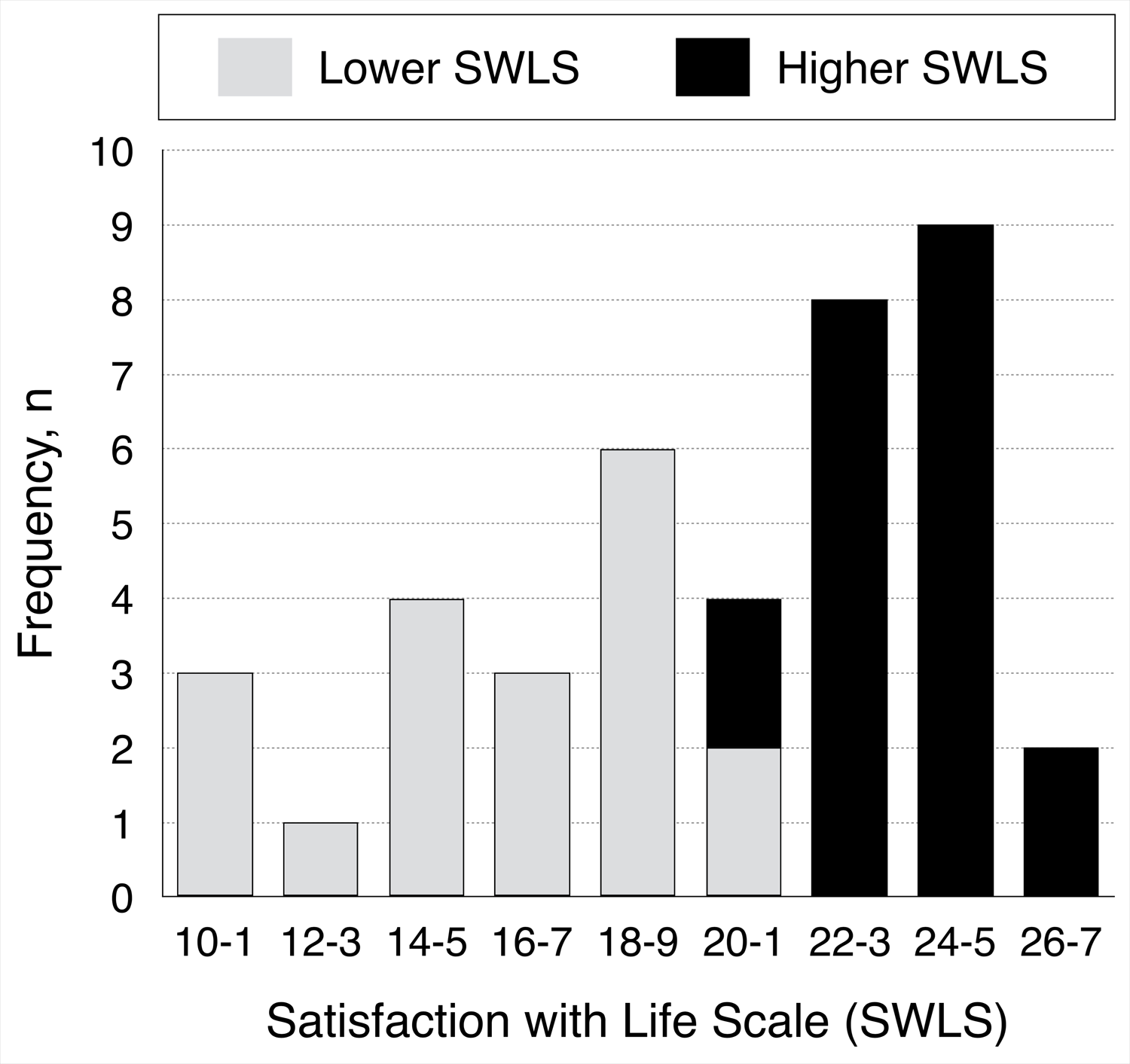

Supplement: S1 Fig — (TIF) [file pone.0149554.s001.tif]
